# Skin-Derived Precursor Cells Promote Angiogenesis and Stimulate Proliferation of Endogenous Neural Stem Cells after Cerebral Infarction

**DOI:** 10.1155/2015/945846

**Published:** 2015-03-26

**Authors:** Duo Mao, Xinpeng Yao, Guowei Feng, Xiaoqing Yang, Lina Mao, Xiaomin Wang, Tingyu Ke, Yongzhe Che, Deling Kong

**Affiliations:** ^1^State Key Laboratory of Medicinal Chemical Biology, Key Laboratory of Bioactive Materials of Ministry of Education, College of Life Sciences, Nankai University, Tianjin 300071, China; ^2^Department of Urology, Second Hospital of Tianjin Medical University, Tianjin Institute of Urology, Tianjin 300211, China; ^3^Department of Endocrinology, Second Affiliated Hospital, Kunming Medical University, Kunming, Yunnan 650101, China; ^4^Department of Anatomy, School of Medicine, Nankai University, Tianjin 300071, China

## Abstract

Stroke is one of the most common diseases that caused high mortality and has become burden to the health care systems. Stem cell transplantation has shown therapeutic effect in ameliorating ischemic damage after cerebral artery occlusion mainly due to their neurogenesis, immune regulation, or effects on the plasticity, proliferation, and survival of host cells. Recent studies demonstrated that skin-derived precursor cells (SKPs) could promote central nervous system regeneration in spinal cord injury model or the neonatal peripheral neuron. Here, we investigated the therapeutic potential of SKPs in a rat model of cerebral ischemia. SKPs were isolated, expanded, and transplanted into rat cortex and striatum after transient middle cerebral artery occlusion. Our results revealed that SKPs transplantation could improve the behavioral measures of neurological deficit. Moreover, immunohistology confirmed that SKPs could secrete basic FGF and VEGF in the ischemic region and further markedly increase the proliferation of endogenous nestin^+^ and *β*III-tubulin^+^ neural stem cells. Furthermore, increased angiogenesis induced by SKPs was observed by vWF and *α*-SMA staining. These data suggest that SKPs induced endogenous neurogenesis and angiogenesis and protected neuron from hypoxic-ischemic environment. In conclusion, SKPs transplantation may be a promising approach in treatment of stroke.

## 1. Introduction

Stroke is a neurodegenerative disorder and a leading cause of death and disability in many countries, but only a few options support efficient recovery in stroke patients. Over the last 20 years, stem cell-based therapies were raising increasing attention on the treatment of stroke [1, 2]. Administration of stem cells resulted in beneficial effects on neuronal survival and recovery after experimental stroke [3, 4]. But limited stem cells for clinical application including ethical and safety concerns associated with the use of pluripotent stem cells have spurred great interest in the search for alternative stem cell sources for stroke treatment.

Autologous stem cells such as bone marrow mesenchymal stem cells (BM-MSCs) appeared as candidate for the regenerative therapy in the stroke treatment [3, 5, 6]. Nevertheless, BM-MSCs must be isolated by bone marrow aspiration, which is traumatic and painful. Moreover, the percentage of stem cells in bone marrow is very low and decreases with age, thus making it difficult to harvest a sufficient number of high-quality cells for clinical application. Recently, skin-derived precursor cells (SKPs) have attracted attention because they are more readily accessible from adult tissue [7] which belong to neural crest cell populations in various locations of the adult organism [8]. SKPs can proliferate and differentiate into subpopulations of cells expressing neuronal, glial, smooth muscle, and adipocyte markers in vitro [7, 9, 10]. Moreover, SKPs show some properties like Schwann-like cells in the process of somatic nerve regeneration [11–13]. However, there has been no study on whether SKPs can effectively recover function of brain after stroke. Meanwhile, the exact effect of transplanted SKPs on damage tissue has not been clarified [14].

Most studies have shown that the underlying mechanisms of functional recovery following autologous stem cells transplantation are likely mediated by the release of growth factors, which promotes endogenous repair mechanisms, rather than stimulates neuronal differentiation or implant integration at the ischemic site [15, 16]. Moreover, neural crest-derived stem cells have been proved to secrete several active factors such as brain-derived neurotrophic factor (BDNF), basic fibroblast growth factor (bFGF), and Glial cell line-derived neurotrophic factor (GDNF) to enhance neurogenesis in brain after stroke [17, 18]. Besides, transplanted SKPs can be easily became functional vascular SMCs [19] and endothelial cells [20, 21] in the healing wound. According to these evidences, we speculated that SKPs might protect neurons, promote angiogenesis and neural regeneration, and further reduce functional deficits following transplantation into lesion areas in a rat model of transient ischemia induced by middle cerebral artery occlusion (MCAO). Our results suggested that SKPs exert multiple, independent effects on the ischemic brain that may modify outcome after stroke.

## 2. Materials and Methods

### 2.1. Isolation, Cultivation, and Characterization of SKPs

Neonatal male Sprague-Dawley rats were obtained from the Laboratory Animal Center of the Academy of Military Medical Sciences (Beijing, China). All experimental procedures were conducted in conformity with institutional guidelines for the Care and Use of Laboratory Animals in Nankai University Animal Care and Use Committee. SKPs were isolated and cultured as described previously [9]. Briefly, SKPs were prepared from infant rat skin (1–3 weeks) which was cut into 2-3 mm^2^ pieces by a sterile razor blade and then transferred into a 50 mL tube containing 1 mg/mL Dispase II and 0.5 mg/mL collagenase I in DMEM/F12 medium for 30 min at 37°C, mechanically dissociated, and filtered through a 40 *μ*m cell strainer. Cells were plated at a density of 1–2.5 × 10^4^ cells/mL in proliferation medium including DMEM/F12 (Gibco) 3 : 1 containing 0.1% penicillin/streptomycin (Invitrogen), 40 ng/mL bFGF (Invitrogen), 20 ng/mL EGF(Invitrogen), and 2% B27 supplement (Invitrogen) at 37°C, 5% CO_2_.

To passage SKPs, floating spheres were mechanically dissociated and reseeded in fresh medium containing B27 and growth factors at a density of 1 × 10^5^ cells/mL. Cells were passaged every 7 days. To characterize the isolated SKPs, we utilized flow cytometry (FACS) method. SKPs single cell suspensions from day 14 were obtained by treatment with 0.5 mg/mL of Dispase II (Roche, Indianapolis) at 37°C for 30 min. Cells were passed through a 40 *μ*m cell strainer and were incubated with triton X-100 for 10 min at RT and then, and cells were incubated for 30 min at 4°C with mouse anti-nestin and anti-*α*-SMA antibody, respectively. After being washed three times, the FITC-conjugated donkey anti-mouse secondary antibodies were added into the cell suspensions and incubated for 1 hour at 4°C. Cells were analyzed using FACScan (BD Pharmingen, San Jose, CA). To determine the differentiation potential of SKPs, myogenic, neurogenic and adipogenic differentiation was induced by medium (DMEM/F12 3 : 1 containing 0.1% penicillin/streptomycin, 2% B27 supplement). Medium changes were carried out every two days. Adipogenic differentiation was assessed by the cellular accumulation of neutral lipid vacuoles after cells were fixed with 4% formaldehyde, stained with Oil red O (Sigma-Aldrich).

To identify undifferentiated or differentiated SKPs, cells were centrifuged at low speed, dissociated, collected, and adhered to a poly-D-lysine substratum overnight. For immunocytochemistry, cells were fixed with 4% paraformaldehyde, washed with PBS, and treated with different antibodies including nestin (Abcam), fibronectin (Santa Cruz), P75 (Abcam), *α*-SMA (Abcam), glial fibrillary acidic protein GFAP (Santa Cruz), and *β*-III tubulin (Santa Cruz). After overnight incubation at 4°C with primary antibodies, cells were incubated at room temperature for 60 min with secondary antibodies. The secondary antibodies included Rhodamine-conjugated donkey anti-mouse, FITC-conjugated anti-rabbit, and anti-goat IgG (Invitrogen). After washing in PBS, samples were counterstained with a mounting medium containing DAPI (Vector Laboratories) and examined by fluorescence microscopy.

### 2.2. Animal Middle Cerebral Artery Occlusion Model

Transient middle cerebral artery occlusion (MCAO) was induced as previously reported [22] with a slight modification. Adult male Sprague-Dawley rats weighing 280–300 g were anesthetized with 5% isoflurane in O_2_ using an induction chamber and maintained at 3% isoflurane using a face mask. Temperature was maintained at 37°C throughout the surgical procedure, using an electronic temperature controller linked to a heating pad. The right common carotid artery (CCA), external carotid artery (ECA), and internal carotid artery (ICA) were exposed through a ventral midline incision. A 4-0 monofilament nylon suture with a rounded tip was introduced into the CCA lumen and gently advanced into the ICA until it blocked the bifurcating origin of the MCA. Reperfusion was accomplished by withdrawing the suture after 60 min of ischemia.

### 2.3. Experimental Groups and Transplantation Procedures

The animal experiments consist of three groups: Group 1: SKPs (0.5 × 10^6^) (*n* = 6); Group 2: Saline (*n* = 5); Group 3: Sham (*n* = 3). The injection operations were performed 24 h after MCAO. Prior to transplantation, SKPs were digested. Particularly, cell spheres were collected by centrifugation and then added 1 mg/mL dispase II enzyme to digest for about 30 min. The spheres were dissociated into single cells through mechanical approach, which were collected by centrifugation. DiI was dissolved in absolute ethanol (2.5 mg/mL) and added to the cell suspension so that the final concentration was 40 *μ*g/mL. Cells were incubated in the DiI-containing medium for 30 minutes at 37°C and then washed three times with PBS. Stereotaxic injections were performed using Hamilton microsyringe with a 26-gauge blunt needle. Each animal received an injection of 10 *μ*L (at the rate of 1 *μ*L/min, and concentration 50000 cells/*μ*L) of DiI-SKPs into the striatum (from bregma: A + 1.0 mm, L + 2.0 mm, V 22.6 mm). The needle was left in situ for 2 min after injection before removal. At day 7 after cell transplantation, rats received injection of BrdU (Sigma, 10 mg/mL in saline) twice a day (50 mg/kg, i.p.) for 7 consecutive days. These rats were sacrificed 14 days after the cells injection [23].

### 2.4. Behavioral Testing

All animals were trained for 1 week after MCAO. And these behavioral measurements were performed every day since induction of MCAO until sacrifice. A modified neurological severity score (mNSS) was used in this study which includes (1) response to raising the rat-tail and placing it on the flat surface; (2) abnormal movement such as immobility, tremor, and seizures; (3) sensory deficit; (4) absent reflexes such pinna, corneal, and startle. Normal score is 0; maximal deficit score is 18 [6].

### 2.5. Histological and Immunohistochemical Assessment

Animals were reanaesthetized with 5% isoflurane in O_2_ 14 days after surgery. Rat brains were fixed by transcardial perfusion with saline, followed by perfusion and immersion in 4% paraformaldehyde, and the brain were embedded in paraffin and cut into 5 *μ*m sections. The area of both hemispheres was measured on eight serial coronal sections per brain (200 *μ*m apart) stained with hematoxylin and eosin, and the infarction area was averaged over these eight levels. The lesion size was estimated as a percentage of the whole brain by using the following formula: [(area of contralateral hemi-sphere) − (area of remaining ipsilateral hemisphere)/(area of contralateral hemisphere) × 100/2]. To identify proliferating cells, samples were incubated in 2 N HCl at 37°C for 30 minutes and then rinsed in 0.1 M boric acid with pH = 8.6. Samples were incubated with primary antibodies against BrdU at 4°C for overnight. After washing with 0.01 M PBS, samples were incubated with secondary antibodies (FITC-labeled polyclonal goat anti-mouse). For a morphological analysis of vessels, a polyclonal antibody against Von Willebrand factor (vWF; Dako) and *α*-SMA (Abcam) was used. The secondary antibodies were Rhodamine-conjugated donkey anti-mouse, anti-rabbit, and anti-goat IgG (Invitrogen). Slides were observed under confocal laser scanning microscopy (Leica TSC SP8, Germany). To investigate which cell-specific makers co-localized with BrdU-positive nucleus, sections were treated with different antibodies including vWF (Abcam), nestin (Abcam), and coimmunostaining with BrdU. To detect neuroblasts, a polyclonal antibody against nestin (Abcam) and *β*-III tubulin (Santa Cruz) were used. In order to quantify the number of immunoreactive cells, three representative sections from each animal were analyzed. The numbers of cells was blindly counted within 0.25 mm^2^ of subventricular zone (SVZ), ischemic border zone (IB), and ischemic zone (IZ) using NIH image software, Image J. The immunoreactive cells were manually marked and calculated with Image J.

### 2.6. Statistical Analysis

All quantitative results were obtained from at least three samples for analysis. Data were expressed as the mean ± SD. An independent *t*-test was used for two group comparisons and one-way ANOVA for multiple-group comparison, with suitable post hoc analysis. The level of statistical significance was set at *P* < 0.05.

## 3. Results

### 3.1. Characterization and Differentiation of SKPs

**Figure 1:**
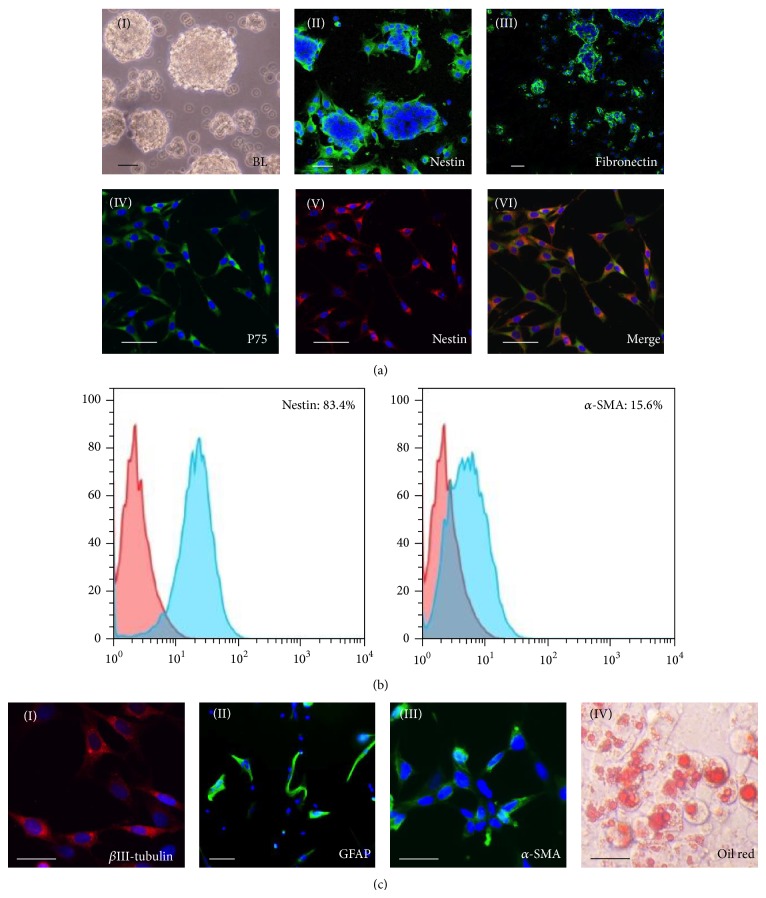
Phenotypic characterization and differentiation of SKPs. ((a)(I)) The appearance of SKPs by phase-contrast microscopy was neurospheres-like before passaging. ((a)(II), (a) (III)) SKPs that were adhered to a poly-d-lysine substratum overnight and then separately labeled with antibodies to nestin and fibronectin. ((a)(IV)–(VI)) Immunofluorescence colocalization analysis of SKPs showed coexpression of nestin (red) and P75 (green), and the nuclei were stained by DAPI. (b) Flow cytometric analysis of cell markers on nestin and *α*-SMA. Percentages indicate the fraction of cells that stained positive. (c) Differentiation of expanded SKPs into neural and mesodermal lineage cells in vitro. SKPs induced method was described in the “Section 2.” ((c)(I)–(III)) Immunostaining for SKPs, *β*III-tubulin (red), the astrocyte marker GFAP (green), and smooth-muscle actin (*α*-SMA; red). ((c)(IV)) Adipogenesis was visualized by staining Oil red O. Scale bars, 10 *μ*m.

As shown in bright field picture (Figure 1(a)(I)), the rat SKPs developed into sphere-like structure in suspension cultures. The SKPs specific marker nestin (Figure 1(a)(II)), fibronectin (Figure 1(a)(III)), neural crest stem cells marker P75 (Figures 1(a)(IV)–1(a)(VI)), and *α*-SMA negative were examined by immunocytochemistry methods and FACS (Figure 1(b)). After induction, SKP spheres began to express neuroblast marker *β*-III tubulin (Figure 1(c)(I)) and astrocyte marker GFAP (Figure 1(c)(II)), indicating the neural potential of SKP cells in vitro. Furthermore, some cells were positively stained for *α*-SMA (Figure 1(c)(III)) and Oil red O (Figure 1(c)(IV)), which revealed the mesodermal cell types differentiation capacity.

### 3.2. Neurobehavioral Tests and Lesion Size

**Figure 2:**
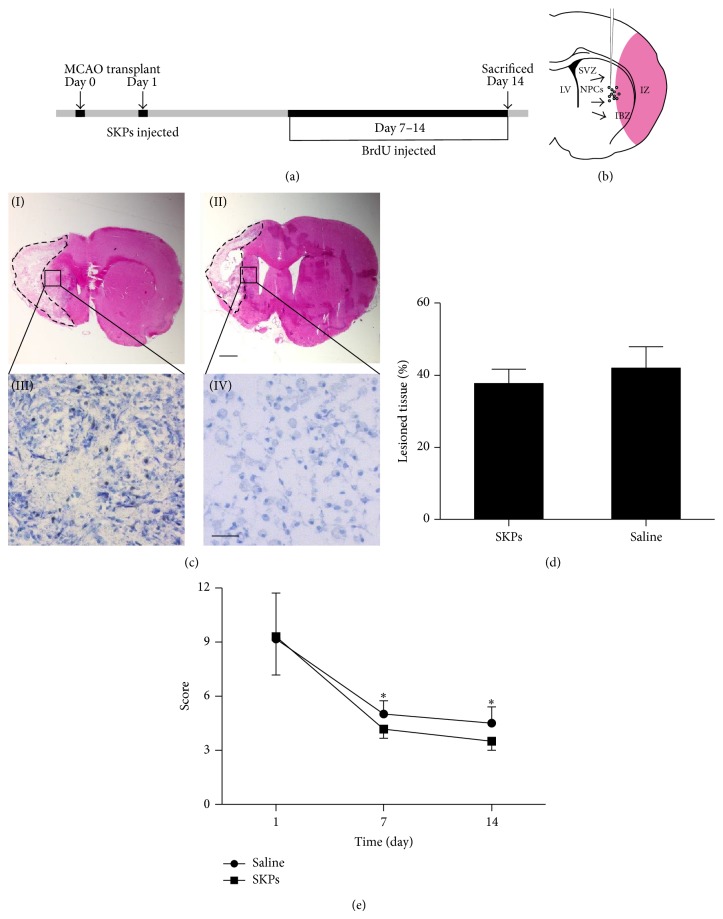
Transplantation of SKPs ameliorates the behavioral impairments and reduces infarct volume in stroke model of rats. (a) Experimental study design. MCAO: middle cerebral artery occlusion; BrdU: bromodeoxyuridine. (b) Representation of the lateral ventricle wall that includes the stem cells injection site, neural progenitor cells (NPCs), the lateral ventricle (LV), the subventricular zone (SVZ), the ischemic boundary zone (IBZ), and the ischemic zone (IZ). ((c)(I), (c)(II)) Representative pictures of HE from animals treated with PBS or SKPs after MCAO. Scale bar, 1 mm. ((c)(III), (c)(IV)) Higher magnification showed that SKPs increased the number of cells with normal neuronal morphology and decreased the number of shrunken and misshapen cells in cresyl violet–stained sections. Scale bar, 10 *μ*m. (d) Infarct size was measured on HE brain sections. Relative infarct size of PBS or SKPs-treated animals is presented as the mean ± S.D. The percentage of lesion tissue in the two groups (SKPs, Saline) at 14 days after occlusion. Two-way ANOVA with repeated measurements followed by one-way ANOVA and post hoc multiple comparison tests using Fisher's PLSD. (e) Behavioral performance in the neurological score (NSS) tests of PBS or SKPs injected animals from 1 to 14 days after ischemia. Statistically significant differences between the SKPs group with PBS group were determined by ANOVA, ^*^ *P* < 0.05.

To study SKPs induced neurogenesis and angiogenesis after cerebral ischemia, rats were subjected to MCAO, given SKPs on day 1 and injected cell proliferation maker BrdU on days 7–14 after ischemia injury (Figure 2(a)). Two weeks after treatment, SKPs transplantation did not result in significant reduction of lesion size compared to the control group by hematoxylin-eosin staining (Figures 2(c)(I) and 2(c)(II)) and statistical analysis (Figure 2(d)). However, the modified neurological severity score (mNSS) was significantly improved at 7 and 14 days in the SKPs treatment group (Figure 2(e)). Compared with control group, toluidine blue staining showed that more neuron exhibited relatively homogenous oval shaped nuclei in SKPs group (Figures 2(c)(III) and 2(c)(IV)). These data suggest that SKPs may contribute to neurological function improvement after stroke.

### 3.3. The Function of SKPs in Rat Brain

**Figure 3:**
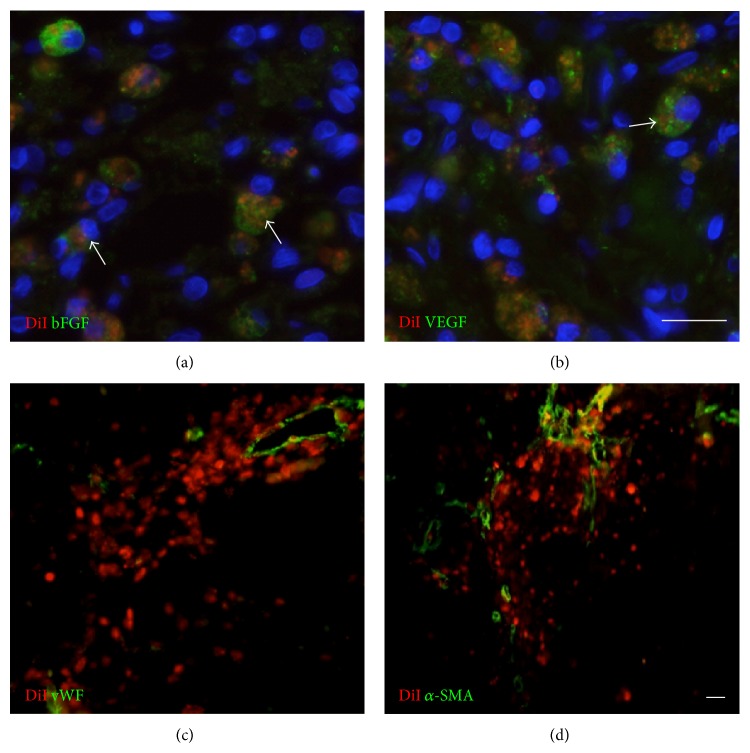
Implanted SKPs secrete growth factors and differentiate into blood vessel in vivo. Immunostaining of DiI and growth factor bFGF, VEGF and blood vessel maker vWF, *α*-SMA in implantation site of IBZ. Brain sections were immunostained for ((a), (b)) bFGF, VEGF (green) or for ((c), (d)) vWF, *α*-SMA (green). Arrowhead shows DiI positive cells (red) that were colocalized with bFGF and VEGF (green). Scale bars, 10 *μ*m.

In order to clarify the therapeutic contribution of SKPs, DiI^+^ SKPs were first immunostained with basic FGF and VEGF. Our results revealed that the transplanted DiI^+^ cells were colocalized with trophic factors bFGF and VEGF (Figures 3(a) and 3(b)). Meanwhile, the higher blood vessel density with marker vWF (Figure 3(c)) and *α*-SMA (Figure 3(d)) were observed around DiI^+^ cells, which indicated that secreted growth factors had angiogenic capacity in the ischemic tissue and could ameliorate the function of brain after injury.

### 3.4. The Neurogenesis and Angiogenesis Effect of Transplanted SKPs

**Figure 4:**
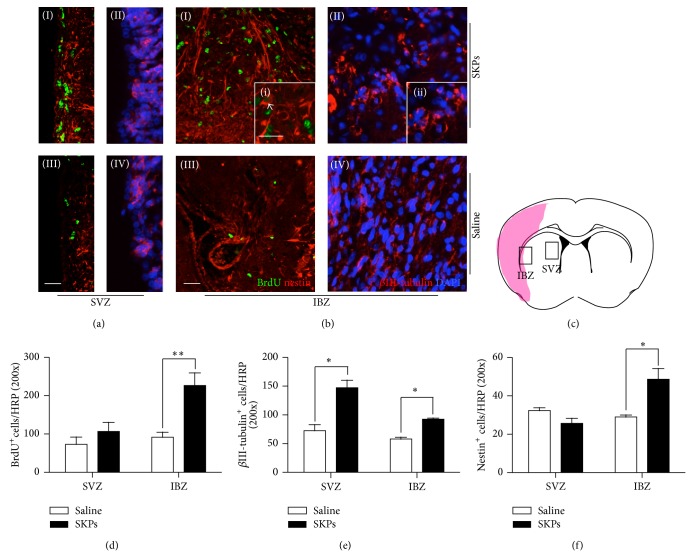
SKPs increase SVZ and IBZ neurogenesis and neuronal progenitor migration to the ischemic lesion 14 days after MCAO. Immunostaining of neural stem cells markers and colocalization of BrdU in (a) SVZ and (b) IBZ of the ischemic rat brain. Brain sections were immunostained for both ((a)(I); (a)(III); (b)(I); (b)(III)) BrdU (green) and nestin (red) or for ((a)(II); (a)(IV); (b)(II); (b)(IV)) *β*III-tubulin in SKPs group (top) and PBS (bottom). ((i), (ii)) Higher magnification of indicated by the white box. Arrowhead shows BrdU positive cells that were colocalized with nestin. Scale bars, 10 *μ*m. (c) The pattern of implanted SKPs after cerebral brain ischemia. SVZ and IBZ were indicated by the black box, shadow area, and infracted zone. Quantitative analysis of (d) BrdU, (e) *β*III-tubulin, and (f) nestin positive cells in the SVZ and IBZ (*n* = 5 for control and SKPs group). ^*^ *P* < 0.05, ^∗∗^ *P* < 0.01 versus control.

To study the proliferation of endogenous stem cells in subventricular zone (SVZ) and ischemic boundary zone (IBZ), animals received BrdU injection for 7 days before sacrifice. Colocalization of BrdU-positive cells with nestin was observed in the SVZ (Figures 4(a)(I) and 4(a)(III)) and IBZ (Figures 4(b)(I) and 4(b)(III)) areas 14 days after stroke. The number of total BrdU^+^ cells had no significant difference between cell therapy group and control group in SVZ (Figure 4(d)). Meanwhile, BrdU^+^ cells also are widely distributed in areas from SVZ to infarct boundary in both groups. Compared with control group, the BrdU^+^ cells had a 2.5-fold increase in IBZ. Moreover, nestin^+^ cells had a 2-fold increase in IBZ of SKPs treated groups, but no significant difference between two groups in SVZ (Figure 4(f)). *β*-III tubulin is a marker that is expressed in newborn neuroblasts and used to trace the nascent cells. At second week after stroke, we observed that *β*-III tubulin^+^ cells are distributed in SVZ (Figures 4(b)(II) and 4(b)(IV)) and IBZ (Figures 4(b)(II) and 4(b)(IV)). Compared with control group, the number of *β*-III tubulin^+^ cells was increased 2-fold in SVZ, 1.4-fold in IBZ of SKPs group (Figure 4(e)). The increased BrdU^+^ cells and migrated neuroblasts indicated ameliorated microenvironment in the infract region which promoted neurogenesis in SVZ.

**Figure 5:**
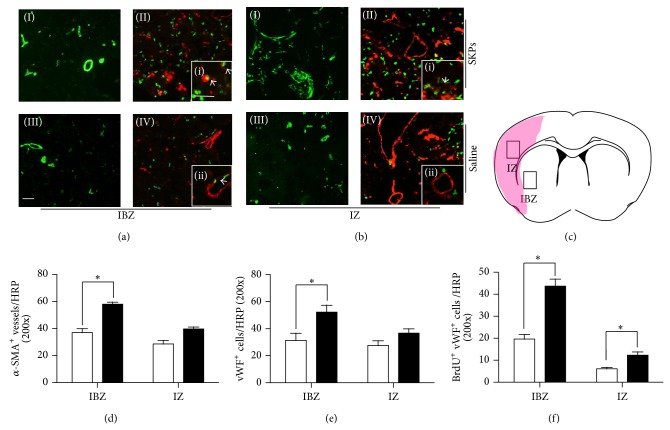
SKPs increase IZ and IBZ revascularization in MCAO model of rats. BrdU immunoreactive endothelial cells, *α*-SMA, and vWF vessels were detected in (a) IBZ and (b) IZ. Brain sections were immunostained for ((a)(I), (a)(III); (b)(I), (b)(III)) *α*-SMA or for ((a)(II), (a)(IV); (b)(II), (b)(IV)) both BrdU (green) and vWF (red) in SKPs group (top) and PBS (bottom). ((i), (ii), (iii), and (iv)) Higher magnification of indicated by the white box. Arrowhead shows BrdU positive cells that were colocalized with vWF. Scale bars, 10 *μ*m. (c) The pattern of implanted SKPs after cerebral brain ischemia. SVZ and IZ were indicated by the black box, shadow area, and infracted zone. Quantitative data of number of (d) vWF or (e) *α*-SMA immunoreactive vessels and (f) BrdU immunoreactive endothelial cells. Injected SKPs (*n* = 5) significantly (*P* < 0.05) increased the number of endothelial cells and the density of vessels in the IZ and IBZ compared with group treated with PBS (*n* = 5).

The number of *α*-SMA^+^, vWF^+^ vessels and vWF^+^/BrdU^+^ cells at 14 days after transplantation in IBZ (Figure 5(a)) and ischemic zone (IZ) (Figure 5(b)) were analyzed by double immunofluorescence staining and visualized by laser scanning confocal microscopy respectively. By counting the number of *α*-SMA^+^ vessels, which represent large blood vessel, the results were significantly higher than that of control group in IBZ but not in IZ (Figure 4(d)). Meanwhile, the vWF^+^ vessel density of SKPs group has followed the same trend (Figure 5(e)). However, the numbers of vWF^+^/BrdU^+^ cells were increased 2.3-fold in IBZ, 2-fold in IZ (Figure 5(f)). These data demonstrate that SKPs have an obvious effect on revascularization of ischemic damage.

## 4. Discussion

In this study, we demonstrated that injection of 0.5 × 10^6^ SKPs (24 h after brain ischemia) significantly improved functional outcome compared with control group at day 7 and day 14. Morphological analysis indicated that paracrine signaling of SKPs played a major role to enhance vessel density, cellular proliferation, and neurogenesis along the lateral ventricle and in the striatal ischemic boundary zone, which likely contributed to the improvement of neurological functional recovery in rat after stroke.

SKPs were initially derived from neural crest and displayed multidirectional differentiation capacity including mesodermal and neural progeny during long-term culture [24]. However, the ability of differentiation into electrophysiologically active neural cells has not been proved by animal model just through hippocampal slices culture [14]. Here, we do not observe any DiI^+^ neural cells differentiation in injection area perhaps due to low cell viability after transplantation. In the present research, the fact that transplanted SKPs improved functional restoration without the reduction of lesion area in the ischemic brain of rats would be more probably due to neurorestorative effects of proteins released by transplanted SKPs, which resulted in neurogenesis and angiogenesis in the ischemic boundary zone.

It was known that NSCs reside in the specific region of brain. When damaged, NSCs will be mobilized and migrate toward injury site immediately in the first two weeks [25] and yet were hard to survive due to low blood-supply level and hypoxia in the local microenvironment [26]. To overcome this problem, MSCs [27] and olfactory ensheathing cells [28] were applied to neuroprotective in stroke animal models of stroke. Noteworthy this is the first report in which the treatment outcome was found in ischemic brain tissue after SKPs transplantation. Results showed that SKPs also secrete VEGF and bFGF, which could help in vascular remolding. Increased vWF-immunoreactive vessel density and the number of BrdU^+^ vWF^+^ cells in the rats treated with SKPs indicated that SKPs modulated vascular system and stimulated endothelial cells proliferation.

Previous study reported that, compared to MSCs, SKPs were able to secrete more neurotrophic molecules (like BDNFs, GDNFs, and bFGF) that exhibit substantial effects on neuron survival and functions [29]. Neurotrophic molecules not only stimulates neurite outgrowth for several neuronal cell types in vitro but also stimulates regrowth of multiple descending axon tracts within the spinal cord following injury [30]. In addition, transplantation of neural stem cells overexpressing GDNF enhanced neurogenesis in rats after stroke [31]. We also demonstrated that treatment with SKPs significantly increased the number of BrdU incorporating cells, nestin-immunoreactive cells, and the *β*-III tubulin-immunoreactive cells in the SVZ suggesting that SKPs treatment enhanced endogenous neurogenesis. Moreover, H&E and toluidine blue staining showed that most neuron exhibited relatively homogenous oval shaped nuclei in SKPs group and less extent of inflammation in SKPs group than the control group. It has been reported that extracellular matrix (ECM) affects cells survival, proliferation, and migration [32]. Fibronectin is a crucial component of the ECM that has been demonstrated to stimulate nerve fiber growth in vitro and exert a neural protective effect after stroke [33]. In our study, SKPs expressed fibronectin in culture. Therefore, it is possible that fibronectin may participate in neuron survival and differentiation and involve the functional restoration by activating integrin signal transduction and reestablishing new neuronal circuits in host brain tissue. Taken together, these data further explain why SKPs could enhance neurological function recovery.

## 5. Conclusion 

Transplantation of SKPs into rat brain after stroke improved neurological function recovery by promoting neurogenesis and neovascularization, because SKPs are readily accessible pluripotent sources and possess various therapeutic capacities, which may become a promising candidate cell source for treatment of stroke.
